# Alpha and Beta Synucleins: From Pathophysiology to Clinical Application as Biomarkers

**DOI:** 10.1002/mds.28941

**Published:** 2022-02-05

**Authors:** Lorenzo Barba, Federico Paolini Paoletti, Giovanni Bellomo, Lorenzo Gaetani, Steffen Halbgebauer, Patrick Oeckl, Markus Otto, Lucilla Parnetti

**Affiliations:** ^1^ Section of Neurology, Laboratory of Clinical Neurochemistry, Department of Medicine and Surgery University of Perugia Perugia Italy; ^2^ Department of Neurology University of Ulm Ulm Germany; ^3^ Department of Neurology Martin‐Luther‐University Halle‐Wittenberg Halle/Saale Germany; ^4^ German Center for Neurodegenerative Disorders Ulm (DZNE e. V.) Ulm Germany

**Keywords:** alpha‐synuclein, beta‐synuclein, cerebrospinal fluid, biomarkers, Parkinson's disease

## Abstract

The synuclein family includes three neuronal proteins, named α‐synuclein, β‐synuclein, and γ‐synuclein, that have peculiar structural features. α‐synuclein is largely known for being a key protein in the pathophysiology of Parkinson's disease (PD) and other synucleinopathies, namely, dementia with Lewy bodies and multisystem atrophy. The role of β‐synuclein and γ‐synuclein is less well understood in terms of physiological functions and potential contribution to human diseases. α‐synuclein has been investigated extensively in both cerebrospinal fluid (CSF) and blood as a potential biomarker for synucleinopathies. Recently, great attention has been also paid to β‐synuclein, whose CSF and blood levels seem to reflect synaptic damage and neurodegeneration independent of the presence of synucleinopathy. In this review, we aim to provide an overview on the pathophysiological roles of the synucleins. Because γ‐synuclein has been poorly investigated in the field of synucleinopathy and its pathophysiological roles are far from being clear, we focus on the interactions between α‐synuclein and β‐synuclein in PD. We also discuss the role of α‐synuclein and β‐synuclein as potential biomarkers to improve the diagnostic characterization of synucleinopathies, thus highlighting their potential application in clinical trials for disease‐modifying therapies. © 2022 The Authors. *Movement Disorders* published by Wiley Periodicals LLC on behalf of International Parkinson and Movement Disorder Society

## Graphical abstract

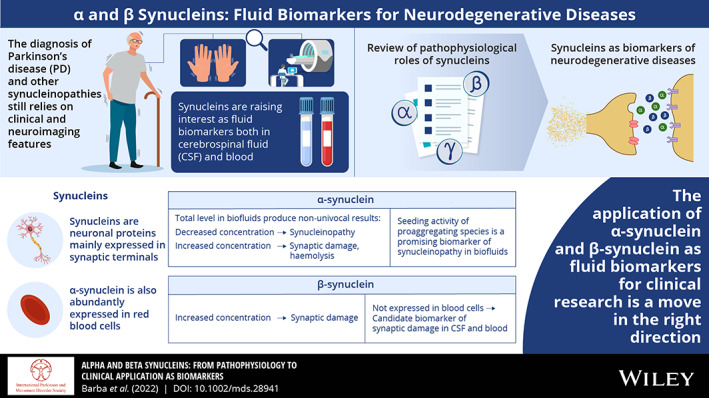

April Infographic : Alpha and Beta Synucleins: From Pathophysiology to Clinical Application as Biomarkers

The synucleins are a family of the following three conserved neuronal proteins: α‐synuclein (140 amino acids), β‐synuclein (134 amino acids), and γ‐synuclein (127 amino acids).^1^ α‐Synuclein has been investigated extensively in neurodegenerative disorders that are referred to as synucleinopathies, including Parkinson's disease (PD), dementia with Lewy bodies (DLB), and multiple system atrophy (MSA).^2^ These disorders share the main pathological feature of accumulation, misfolding, and aggregation of α‐synuclein, which progressively leads to large intracellular aggregates, namely, Lewy bodies and glial cytoplasmic inclusions. Although α‐synuclein is relatively well characterized, the relevance of the two other synucleins for physiological neuronal functioning, as well as their potential contribution to neurodegeneration in synucleinopathies, is still poorly understood. At a clinical level, several studies have focused on the possible application of α‐synuclein as a candidate fluid biomarker for synucleinopathies. The interest is also growing for β‐synuclein, which has been recently measured in cerebrospinal fluid (CSF) and blood from patients affected by synucleinopathies and other neurodegenerative disorders. In this scenario, a major question arises: How can our understanding of synuclein pathophysiology impact the applicability of these proteins as body fluid biomarkers?

In this review, we first summarize the recent evidence of the pathophysiological roles of synucleins. Because the physiological roles of γ‐synuclein, as well as its involvement in neurodegeneration, are far from being clear, we focus on α‐synuclein and β‐synuclein. Second, we discuss the role of α‐synuclein and β‐synuclein as potential biomarkers to improve the diagnostic characterization of patients affected by synucleinopathies. Their potential use in research settings, that is, clinical trials for novel disease‐modifying treatments targeting α‐synuclein accumulation and misfolding, is also discussed.

## 1 Distribution and Structural Features of Synucleins

Synucleins are mainly expressed in the central nervous system (CNS) and encoded by the *SNCA* (α‐synuclein), *SNCB* (β‐synuclein), and *SNCG* (γ‐synuclein) genes.^3^ α‐Synuclein is abundant in neurons, where it represents 1% of all cytosolic proteins,^4^ but other sites of expression are identified in glial and blood cells.^5^ In neurons, α‐synuclein and β‐synuclein colocalize physiologically in presynaptic terminals,^6^ but with aging, α‐synuclein may redistribute to the neuronal soma with a relative decrease in synaptic levels.^7^ The production of α‐synuclein is greatly induced during CNS development, when the protein promotes synaptogenesis and maturation of neuronal precursors.^8^, ^9^ β‐Synuclein is likewise largely expressed in the CNS and retina, whereas γ‐synuclein expression mainly occurs in retinal and peripheral neurons.^10^, ^11^, ^12^

Synucleins are usually defined as intrinsically disordered proteins, and their structural peculiarities largely influence their pathophysiological activities.^13^ The amino acid sequence of synucleins is distinguished into three main regions: N‐terminus, nonamyloid component (NAC) region, and C‐terminus. The N‐terminus is highly conserved among the three synucleins and is responsible for their lipid‐binding properties.^14^ When bound to lipids, the N‐terminal domain assumes an α‐helical structure. The N‐terminal helicity negatively correlates with aggregation potential. It progressively decreases from β‐synuclein and γ‐synuclein to α‐synuclein and is even lower in PD‐associated α‐synuclein mutants.^15^, ^16^ The central NAC region is remarkably aggregation prone in α‐synuclein and constitutes the cross‐β‐sheet motifs within pathological aggregates.^13^ By contrast, the NAC region of β‐synuclein has a central deletion of 11 amino acids that greatly decreases its tendency to aggregate.^17^ The C‐terminal domain profoundly differs among synucleins and regulates their solubility depending on its length and charge.^18^ In particular, the high C‐terminal flexibility of α‐synuclein enhances its propensity to aggregate, whereas β‐synuclein is substantially more rigid as a result of a polyproline secondary structure.^16^, ^19^ The P123H β‐synuclein mutation, which causes familial DLB clusters, seems to confer to β‐synuclein an α‐synuclein‐like behavior by increasing the flexibility of its C‐terminus.^20^, ^21^ In comparison, γ‐synuclein has a relatively shorter C‐terminal domain with fewer acidic residues.^22^

In the cytosolic environment, the physiological forms of α‐synuclein are still not adequately defined because α‐synuclein can be found alternatively in multimeric and monomeric species.^13^, ^23^, ^24^ However, these findings have not been widely replicated. Several cytosolic species of α‐synuclein could be in dynamic equilibrium between free and membrane‐bound states. A transient multimerization process would be therefore crucial for the functioning of α‐synuclein and, if compromised, might lead to pathological aggregation.

Intriguingly, despite being highly conserved neuronal proteins, knock‐out (KO) of a single synuclein does not induce severe alterations in overall neuronal wellness in animal models. α‐Synuclein KO mice experienced only moderate synaptic dysfunction, especially in nigral dopaminergic neurons^25^, ^26^, ^27^ and, surprisingly, double α‐synuclein/β‐synuclein^28^ or α‐synuclein/γ‐synuclein KO mice^29^ show similar and not worse alterations. Only triple‐synuclein KO models have a severe motor phenotype with large synaptic degeneration and early mortality, which could be rescued by overexpression of monomeric α‐synuclein.^30^

## 2 Physiological Roles of Synucleins

**FIG 1:**
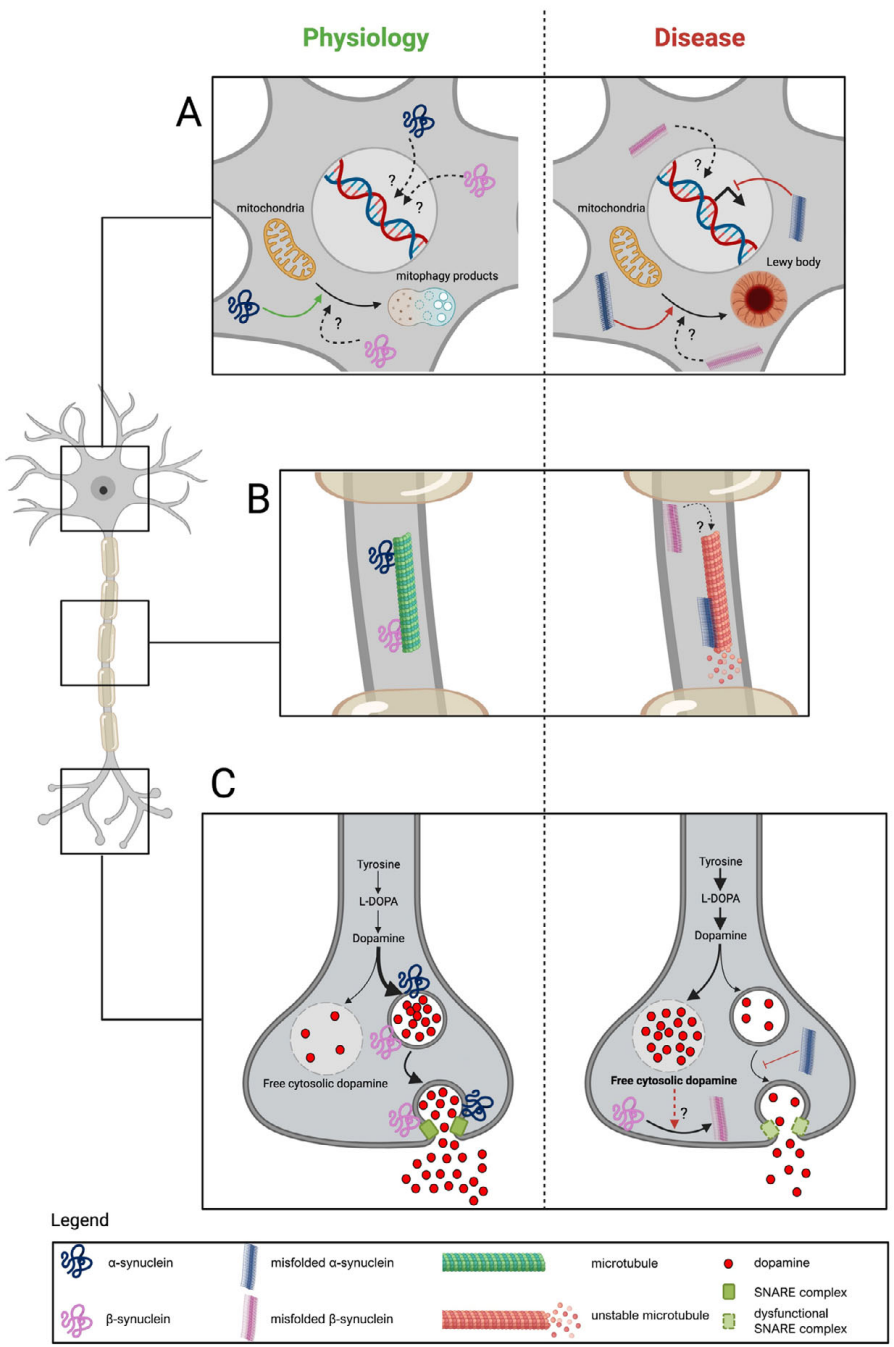
Main pathophysiological roles of α‐synuclein (blue) and β‐synuclein (purple) in neurons. (**A**) In the neuronal body, α‐synuclein participates in mitochondrial functioning and motility, contributing to redirecting damaged mitochondria to mitophagy. When this pathway is impaired, misfolded α‐synuclein may induce accumulation of dysmorphic organelles (ie, mitochondria) and formation of neuronal inclusions. The physiological impact of α‐synuclein on nuclear functions is unclear, whereas pathological α‐synuclein might be related to alterations in the transcription process. To what extent β‐synuclein and misfolded β‐synuclein affect neuronal organelles and nuclei is currently unknown. (**B**) α‐Synuclein interacts with several cytoskeletal components, among which the axonal microtubules are destabilized by misfolded and aggregated α‐synuclein. It is unknown whether β‐synuclein interacts with microtubules and other structural proteins. (**C**) The most relevant functions of α‐synuclein are exerted in presynaptic terminals and mainly deal with regulation of vesicular trafficking and homeostasis of neurotransmitters in both dopaminergic neurons and other cell types. Physiologically, α‐synuclein promotes storage of dopamine into presynaptic vesicles and its release through exocytosis. In synucleinopathies, the synthesis of dopamine might be overstimulated, resulting in dopamine‐induced neurotoxicity. β‐Synuclein may act as a synaptic chaperone and, hypothetically, its aggregation might be promoted by dopamine dyshomeostasis, thus contributing to neuronal damage. L‐DOPA, levodopa; SNARE, soluble N‐ethylmaleimide‐sensitive factor attachment proteins receptors. [Color figure can be viewed at wileyonlinelibrary.com]

α‐Synuclein exerts several physiological functions in neurons, mainly as a key regulator of vesicular trafficking and neurotransmitter exocytosis (Fig. 1). Its most important function is to participate in vesicular dynamics by assisting the assembly of the soluble N‐ethylmaleimide‐sensitive factor attachment proteins receptors (SNARE), which make up a pivotal macromolecular complex of synaptic terminals.^31^ Moreover, α‐synuclein actively modulates both the shape and the dimension of presynaptic vesicles by directly binding their lipid surface.^32^ In dopaminergic neurons, α‐synuclein acts as a central regulator of dopamine (DA) metabolism by inhibiting DA synthesis^33^, ^34^ and inducing both storage in presynaptic vesicles^35^ and extracellular reuptake of the neurotransmitter.^36^ Inside presynaptic vesicles, DA colocalizes with antioxidants,^37^ while free cytosolic DA can undergo oxidative reactions that release toxic DA‐derived compounds, such as DA–quinone, aminochrome, and 5,6‐indole–quinone.^38^ Some of these products can even interact with β‐synuclein and promote its aggregation.^39^ Indeed, by regulating DA synthesis and storage, α‐synuclein might contribute to protect dopaminergic neurons from oxidation‐induced toxicity. The regulatory activities of α‐synuclein on synapse homeostasis might also be of great relevance in other neurotransmitter systems, which have been only partially examined, that is, other monoaminergic centers of the brain.

In addition to synapses, α‐synuclein plays significant physiological roles in other cellular organelles. Mitochondria have a well‐established role in PD pathology^2^ and might be associated with Lewy body formation.^40^ α‐Synuclein participates in mitochondrial energetics by directly interacting with several mitochondrial proteins, such as the respiratory chain complexes^41^ and adenosine triphosphate (ATP) synthase.^42^ Thus, it is not surprising that both α‐synuclein deficiency and α‐synuclein overexpression affect ATP levels and predispose to energy crises.^42^ Intriguingly, α‐synuclein is also involved in mitochondrial motility and mitophagy by promoting the expression of Miro proteins, which are anchoring proteins that connect mitochondria to microtubules and redirect mitochondria to mitophagy when damaged.^43^ Indeed, accumulation of α‐synuclein induces retention of Miro proteins on the mitochondrial surface and delays mitophagy of damaged mitochondria^43^ (Fig. 1). α‐Synuclein interacts with several cytoskeletal components, namely, actin, spectrins, tau, and microtubules. In physiological conditions, α‐synuclein sequesters actin monomers and inhibits their polymerization.^44^ Spectrins are peculiar cytoskeletal partners of α‐synuclein. β‐Spectrin, together with α‐synuclein, physiologically regulates vesicular dynamics in presynaptic terminals.^45^ In contrast, α‐spectrin binds only to pathological α‐synuclein species and promotes cytoskeleton instability.^46^ In microtubules, the activity of α‐synuclein is similar to that of dynamases, and by binding directly to free and assembled tubulin, α‐synuclein influences its polymerazion rate.^47^, ^48^, ^49^ Morever, α‐synuclein and tubulin compete for the same binding domain on tau protein,^50^ a microtubule‐associated protein related to the pathophysiology of Alzheimer's disease (AD) and other neurodegenerative disorders that are referred to as tauopathies: by promoting tau phosphorylation, α‐synuclein may inhibit the stabilizing action exerted by tau on microtubules.^51^, ^52^ Although nuclear α‐synuclein species are of great research interest, the pathophysiological relationship between α‐synuclein and the nucleus is still unclear, especially because there are methodological issues affecting the detection of α‐synuclein in the nuclear environment.^53^ Inside this organelle, α‐synuclein is mainly found in its S129‐phosphorylated form (p‐α‐synuclein),^54^ which is a posttranslationally modified species frequently found in pathological aggregates.^55^ α‐Synuclein can bind directly to more than 300 different regions of DNA and may downregulate the transcription of a variety of genes, mainly involved in cell cycle control and DNA repair systems.^54^, ^56^, ^57^ α‐Synuclein also interplays with epigenetic targets, such as minichromosome maintenance proteins and histones.^56^, ^58^, ^59^

Most studies of the roles of α‐synuclein in neuronal physiology have focused on dopaminergic neurons, as they are frequently affected by α‐synuclein misfolding pathologies. However, it should be further clarified whether and to what extent α‐synuclein affects the general functioning of central neurons, such as dopaminergic cells, but also peripheral neuronal groups, for example, the myoenteric cholinergic neurons of the gut.^60^, ^61^

Interestingly, α‐synuclein takes part in the physiology of nonneuronal cells, particularly blood cells, where it is found in high quantities in erythrocytes, leukocytes, and platelets. Erythrocytes are the main peripheral source of α‐synuclein and account for 99% of α‐synuclein in blood.^5^ In mature erythrocytes, α‐synuclein exerts both structural functions, by tethering cytoskeletal proteins and the cytoplasmic membrane,^62^ and metabolic activities, by regulating iron homeostasis.^63^, ^64^ In animal models, blood α‐synuclein concentrations correlate with hematocrit and hemoglobin levels, and α‐synuclein KO mice experience iron deficiency and moderate anemia.^65^, ^66^ Furthermore, α‐synuclein may play a significant role during hematopoiesis because erythroid precursors produce α‐synuclein during maturation.^67^ Platelets and leukocytes similarly rely on α‐synuclein for their physiological functioning, mainly depending on α‐synuclein‐mediated assembly of SNARE complexes, which are also important in these cells.^68^, ^69^

β‐Synuclein and γ‐synuclein are expressed in retinal neurons and have been associated with glaucoma‐related neurodegeneration.^70^, ^71^ To date, no convincing hypothesis has been proposed for the roles of β‐synuclein in neurons, but it may act as a general synaptic chaperone.^72^ In adrenal chromaffin cells, it regulates vesicle dynamics and secretory rate with mechanisms similar to those occurring in neurons.^73^ As opposed to α‐synuclein, β‐synuclein is not expressed in blood cells, thus peripheral β‐synuclein might exclusively derive from brain neurons. Two main homeostatic functions are attributed to γ‐synuclein in neurons and other cell types. First, γ‐synuclein regulates cytoskeletal remodeling during the cell‐cycle,^74^ which has been linked to development and progression of brain tumors and peripheral carcinomas.^70^, ^75^ In addition, γ‐synuclein exerts metabolic activities during the feed–fast cycle in hypothalamus and adipose tissue.^76^

## 3 Synucleins in Neurological Diseases

Misfolding and accumulation of α‐synuclein leads to formation of neuronal inclusions in PD and DLB and of oligodendrocytic bodies in MSA. α‐Synuclein aggregation is a multistep process that is thought to progress from accumulation of monomeric α‐synuclein to nucleation of small soluble oligomers and to formation of large insoluble fibrils. Soluble oligomeric species of α‐synuclein are reported to be more neurotoxic than large fibrils, which may instead have protective effects by sequestering free α‐synuclein monomers.^2^, ^77^ However, recent findings have pointed out that α‐synuclein fibrils may also be a source of toxic soluble oligomers.^78^ Degradation of α‐synuclein species depends mainly on the autophagic lysosomal pathway (ALP) and, to a lesser extent, on the ubiquitin–proteasome system. ALP dysfunctions are key biological features of synucleinopathies and lead to multiorganelle toxicity as both a cause and a consequence of α‐synuclein aggregation.^79^ Accumulation of damaged and dysmorphic organelles that are improperly cleared in case of ALP dysfunction (eg, mitochondria) may contribute to the formation of Lewy bodies being stuck and tethered together by α‐synuclein molecules.^40^

Among the factors that modulate the dynamics of α‐synuclein aggregation,^35^ β‐synuclein deserves to be mentioned. It has been shown that β‐synuclein can act as an antiaggregation agent against α‐synuclein and even more efficiently against amyloid‐β peptides by directly binding monomers or early‐stage oligomers and preventing them from further growth.^80^, ^81^, ^82^ Nevertheless, the apparent protective action of β‐synuclein on protein misfolding should not be misinterpreted because, in certain conditions, β‐synuclein behaves rather as an amyloidogenic protein and contributes to neurodegeneration.^39^, ^83^ Indeed, the antiaggregation properties of β‐synuclein occur only when α‐synuclein and β‐synuclein are coexpressed at fixed ratios^84^ while at acidic pH, a condition that can be found in lysosomal compartments of the cell, α‐synuclein and β‐synuclein aggregate at similar rates.^85^ Rather than being a proper antiaggregation agent, β‐synuclein could be a less aggregation‐prone protein that appears beneficial only when compared with proteins with a higher amyloidogenic potential, such as α‐synuclein and amyloid‐β peptides. β‐Synuclein could thus express its pathological potential only in particular conditions. Indeed, overexpression of α‐synuclein or β‐synuclein produces similar pathological features in preclinical models, mainly associated with ALP dysfunction^72^, ^79^ and mitochondrial impairment.^39^ However, α‐synuclein pathology determines an overall more severe phenotype and uniquely impacts mitochondrial motility.^43^, ^84^

Although α‐synuclein pathology may affect several cerebral regions and lead to widespread neurodegeneration,^86^ dopaminergic neurons show some unique vulnerability factors. This vulnerability might be partially related to DA dyshomeostasis because DA and DA‐derived products are able to promote aggregation of both α‐synuclein and β‐synuclein, especially in the presence of an excessive oxidative burden.^39^, ^84^ Moreover, it is still not clear whether the presence of neuromelanin, a pigment uniquely expressed in nigral neurons among the dopaminergic centers of the brain,^37^, ^38^ has a role in α‐synuclein pathology. Indeed, neuromelanin derives from DA metabolites and was found to boost the production of α‐synuclein.^87^ α‐Synuclein, DA, and neuromelanin, together with β‐synuclein, might thus contribute to the selective vulnerability of nigral dopaminergic neurons in synucleinopathies.

Another issue not yet fully clarified concerns the possible involvement of β‐synuclein and γ‐synuclein in synucleinopathies and other neurological disorders. β‐Synuclein‐positive inclusions have been observed in PD and DLB hippocampi^88^ as well as in MSA cerebellum.^89^ Interestingly, β‐synuclein has also been associated with neuroinflammatory disorders because anti‐β‐synuclein immunity drives the gray matter damage in progressive forms of multiple sclerosis.^90^ In addition to their typical neuropathological picture, synucleinopathies,^72^ AD,^91^ brain tumors,^75^ and amyotrophic lateral sclerosis^92^ show γ‐synuclein‐positive inclusions. All of these findings suggest a possible relationship between synucleins and a wide range of neurological conditions.

## 4 α‐Synuclein as Biomarker: State of the Art and Future Perspectives

The diagnosis of PD and other synucleinopathies still relies on clinical and imaging criteria.^93^, ^94^ However, given the frequency of incorrect and delayed diagnosis even when these criteria are properly applied,^95^ the diagnostic work‐up of synucleinopathies is moving toward a molecular‐based assessment, following the route traced by AD.^96^ As the most relevant protein in synucleinopathies, α‐synuclein has been proposed to be a fluid and neuroimaging biomarker for synucleinopathies. Attempts to target α‐synuclein pathology with neuroimaging biomarkers have so far met with limited success. The main problems in α‐synuclein‐targeting compounds include the lack of specificity and the difficulty of crossing the blood–brain barrier (BBB). On one hand, polyaromatic molecules able to cross the BBB and bind α‐synuclein aggregates have shown cross‐reactivity with amyloid‐β plaques.^97^ On the other hand, specific antibodies to α‐synuclein aggregates do not adequately cross the BBB when administered intravenously.^98^ Finally, peptides and antibodies labeled with positron emission tomography and single‐photon emission computed tomography tracers are not suitable agents given the short half life of the isotopes of choice.^98^ Hence, brain imaging for synucleinopathies cannot directly picture α‐synuclein pathology to date but can only demonstrate the structural and functional consequences.

Most of the contributions to the diagnostic characterization of patients with synucleinopathies derive from research on fluid biomarkers, especially in the CSF. α‐Synuclein has been extensively investigated as a candidate fluid biomarker for synucleinopathies because it is a central protein in the pathophysiology of these disorders.^99^ As assessed in several investigations and meta‐analyses, CSF total α‐synuclein (t‐α‐synuclein) concentrations have shown an overall slight decrease in patients with PD compared with healthy and neurological controls.^100^, ^101^, ^102^, ^103^, ^104^, ^105^ Similar findings were found in DLB and MSA,^106^, ^107^ with no differences among synucleinopathies except for one meta‐analysis that showed lower values in MSA compared with PD.^101^ These findings indicate that CSF α‐synuclein may be a possible biomarker of synucleinopathy (Table 1). However, lower α‐synuclein concentrations were also reported in other conditions likely not related to α‐synuclein misfolding, such as corticobasal syndrome and vascular parkinsonism, thus limiting the utility of CSF t‐α‐synuclein for discriminating PD from the atypical parkinsonisms.^103^

**TABLE 1:** Investigations of β‐synuclein and α‐synuclein as diagnostic biomarkers in CSF and peripheral blood and as outcome measures in clinical trials for synucleinopathies

Candidate biomarker	Reference	Species	Method	Cohort	Main findings
CSF β‐synuclein	Oeckl et al 2016^108^	β‐synuclein	MS	30 Controls, 19 PD, 13 PDD, 6 DLB, 18 AD, 6 CJD, 15 PSP, 7 CBS	↑ β‐synuclein AD vs. controls ↑ β‐synuclein in CJD vs. all other groups
	Oeckl et al 2020^134^	β‐synuclein	MS	110 Controls, 25 PD, 13 PDD/DLB, 145 AD, 25 CJD, 15 bvFTD, 30 ALS	↑ β‐synuclein in AD vs. controls ↑ β‐synuclein in CJD vs. all other groups
	Halbgebauer et al 2020^135^	β‐synuclein	ELISA	60 Controls, 46 LBD, 151 AD, 23 CJD, 29 bvFTD, 18 ALS	↑ β‐synuclein in AD vs. controls ↑ β‐synuclein in CJD vs. all other groups
Blood β‐synuclein	Oeckl et al 2020^134^	β‐synuclein	MS	93 Controls, 25 PD, 13 PDD/DLB, 136 AD, 25 CJD, 10 bvFTD, 29 ALS	↑ β‐synuclein in AD vs. controls ↑ β‐synuclein in CJD vs. all other groups
CSF α‐synuclein	Eusebi et al 2017^103^	t‐α‐synuclein, o‐α‐synuclein, p‐α‐synuclein	ELISA, Luminex, TR‐FRET, electro‐chemiluminescence	Meta‐analysis of 34 studies with a total of 1428 controls, 2070 PD, 396 DLB, 309 MSA, 259 PSP, 55 CBS, 22 VaP	↓ t‐α‐synuclein, ↑ o‐α‐synuclein and ↑ p‐α‐synuclein in PD vs. controls = t‐α‐synuclein in PD, DLB, MSA, PSP, CBS, and VaP
	Sako et al 2014^102^	t‐α‐synuclein	ELISA, Luminex, TR‐FRET	Meta‐analysis of 9 studies with a total of 537 controls, 843 PD, 130 MSA, 98 PSP	↓ t‐α‐synuclein in PD vs. PSP and controls ↓ t‐α‐synuclein in MSA vs. PSP and controls = t‐α‐synuclein in PD and MSA
	Zhou et al 2015^101^	t‐α‐synuclein o‐α‐synuclein	ELISA, Luminex, TR‐FRET	Meta‐analysis of 12 studies with a total of 783 Controls, 1131 PD, 192 DLB, 179 MSA, 92 PSP	↓ t‐α‐synuclein in PD vs. controls ↓ t‐α‐synuclein in MSA vs. PD = t‐α‐synuclein in PD and DLB ↑ o‐α‐synuclein in PD vs. controls
	Gao et al 2015^100^	t‐α‐synuclein	ELISA, Luminex, MS, TR‐FRET	Meta‐analysis of 5 studies with a total of 399 controls, 412 PD, DLB, 148 AD, 31 FTD, 32 MSA	↓ t‐α‐synuclein in PD vs. controls = t‐α‐synuclein in PD, DLB and MSA
Blood α‐synuclein	Besong‐Agbo et al 2013^143^	t‐α‐synuclein	ELISA	46 Controls, 34 PD, 42 AD	= t‐α‐synuclein in PD, AD and controls
	Foulds et al 2013^120^	t‐α‐synuclein p‐α‐synuclein	ELISA	91 Controls, 189 PD	= t‐α‐synuclein in PD and controls ↑ p‐α‐synuclein in PD vs. controls
	Shi et al 2014^144^	t‐α‐synuclein Exosomal α‐synuclein	Luminex	215 Controls, 267 PD	= t‐α‐synuclein in PD and controls ↑ Exosomal α‐synuclein in PD vs. controls
	Ishii et al 2015^145^	t‐α‐synuclein	ELISA	103 Controls, 53 PD	↓ t‐α‐synuclein in PD vs. controls
	Williams et al 2016^119^	o‐α‐synuclein	ELISA	5 controls, 9 PD, 6 AD	↑ o‐α‐synuclein in PD vs. AD and controls
	Ding et al 2017^146^	t‐α‐synuclein	ELISA	23 Controls, 84 PD	↑ t‐α‐synuclein in PD vs. controls
	Lin et al 2017^147^	t‐α‐synuclein	IMR	34 Controls, 80 PD	↑ t‐α‐synuclein in PD vs. controls ↑ t‐α‐synuclein in PDD vs. PD
	Vicente Miranda et al 2017^123^	Y125‐p‐α‐synuclein Y39‐n‐α‐synuclein Glycated α‐synuclein SUMOylated α‐synuclein	Immunoblotting and densitometry	30 Controls, 58 PD	↑ Y125‐p‐α‐synuclein, Y39‐n‐α‐synuclein and glycated α‐synuclein, and ↓ SUMOylated α‐synuclein in PD vs. controls
	Malec‐Litwinowicz et al 2018^148^	t‐α‐synuclein	ELISA	38 Controls, 58 PD	= t‐α‐synuclein in PD and controls
	Abd Elhadi et al 2019^121^	t‐α‐synuclein p‐α‐synuclein	ELISA	45 Controls, 32 PD, 14 PDD	↑ t‐α‐synuclein in PD vs. PDD and controls ↑ p‐α‐synuclein in PD vs. controls
	Chang et al 2020^149^	t‐α‐synuclein	IMR	40 Controls, 48 PD	↑ t‐α‐synuclein in PD vs. controls
	Li et al 2021^122^	Erythrocytic p‐α‐synuclein	ELISA	334 Controls, 333 PD	↑ p‐α‐synuclein in PD vs. controls

	Reference	Species	Drug candidate	Cohort	Main findings
Clinical trials	Pagan et al 2016^140^	CSF t‐α‐synuclein	Nilotinib	12 PDD/DLB	↓ t‐α‐synuclein after 2 and 6 months with 150‐mg nilotinib
	Pagan et al 2019^141^	CSF t‐α‐synuclein, CSF o‐α‐synuclein	Nilotinib	75 PD	= t‐α‐synuclein from 1 to 4 hours after drug administration ↓ o‐α‐synuclein 3 hours after administration of 400‐mg nilotinib
	Shin et al 2019^142^	CSF o‐α‐synuclein, plasma o‐α‐synuclein	KM‐819	88 Healthy volunteers	= CSF and plasma o‐α‐synuclein 7 days after drug administration
	NCT03888222	Plasma t‐α‐synuclein, plasma o‐α‐synuclein	Bosutinib	30 DLB	Ongoing
	NCT03996460	CSF and plasma t‐α‐synuclein, CSF and plasma o‐α‐synuclein	K0706	45 DLB	Ongoing

Abbreviations: AD, Alzheimer disease; ALS, amyotrophic lateral sclerosis; bvFTD, behavioral variant frontotemporal dementia; CBS, corticobasal syndrome; CJD, Creutzfeldt‐Jakob disease; CSF, cerebrospinal fluid; DLB, dementia with Lewy bodies; ELISA, enzyme‐linked immunosorbent assay; IMR, immunomagnetic reduction; LBD, Lewy body disorders; MS, mass spectrometry; MSA, multiple system atrophy; o‐α‐synuclein, oligomeric α‐synuclein; PD, Parkinson's disease; PDD, Parkinson's disease with dementia; PSP, progressive supranuclear palsy; t‐α‐synuclein, total α‐synuclein; p‐α‐synuclein, phosphorylated α‐synuclein at serine 129; TR‐FRET, time‐resolved fluorescence energy transfer; VaP, vascular parkinsonism; SUMO, small ubiquitin‐like modifier; Y125‐p‐α‐synuclein, phosphorylated α‐synuclein at tyrosine 125; Y39‐n‐α‐synuclein, nitrated α‐synuclein at tyrosine 39.

Increased CSF t‐α‐synuclein levels were observed in AD and Creutzfeldt‐Jakob disease (CJD),^106^, ^108^, ^109^, ^110^ and, variably, even in progressive supranuclear palsy.^101^, ^102^, ^103^, ^111^ These findings suggest a possible further role of α‐synuclein as a marker of synaptic damage when its concentrations increase by being released from degenerating synapses. The two components of α‐synuclein aggregation and synaptic derangement could variably influence α‐synuclein measurements and partially justify the conflicting results reported. In this regard, longitudinal studies in PD have found increasing CSF t‐α‐synuclein concentrations during the disease course and described associations of t‐α‐synuclein with disease duration, total tau levels, and clinical motor scores.^112^, ^113^, ^114^ Higher t‐α‐synuclein values at baseline correlated with faster motor decline,^113^ whereas the predictive value of α‐synuclein for cognitive decline in synucleinopathies has been inconsistent.^99^ A clear‐cut interpretation is challenging because these studies have largely not been replicated^115^ and have shown some methodological concerns, such as the suboptimal CSF sample storage and the lack of standardized procedures. Moreover, the possible mechanisms underlying the longitudinal increase of t‐α‐synuclein in synucleinopathies, as well as its real meaning on a prognostic level, if it has any, are not yet clarified.

Together with t‐α‐synuclein concentrations, pathological species of α‐synuclein, including S129‐phosphorylated (p‐α‐synuclein), oligomeric (o‐α‐synuclein), and proaggregating forms, have been tested as potential diagnostic and prognostic biomarkers because they may more precisely identify the underlying synucleinopathy.^116^ CSF p‐α‐synuclein and o‐α‐synuclein levels were reported to be higher in PD than in controls,^101^, ^103^ but their diagnostic accuracy, when considered singularly, is unsatisfactory for use in clinical practice.^117^ Better performances were instead obtained with combinations of α‐synuclein species and other biomarkers, such as the o‐α‐synuclein/t‐α‐synuclein ratio^117^ and the panel consisting of p‐α‐synuclein, o‐α‐synuclein/t‐α‐synuclein ratio, and phosphorylated tau at threonine 181.^118^

Investigations of α‐synuclein in peripheral blood have shown conflicting results, and the diagnostic utility of blood α‐synuclein species for synucleinopathies should be further assessed before considering any routine clinical use. The main hampering issues concern data reproducibility and preanalytical confounders, such as the release of α‐synuclein from blood cells.^99^ Plasma and serum t‐α‐synuclein levels were reported to be either higher, lower, or not significantly altered in patients with PD compared with controls, whereas more concordant results were provided by assessing pathological forms of α‐synuclein (Table 1). Increased values of p‐α‐synuclein and o‐α‐synuclein were found in the blood and erythrocytes of patients with PD compared with controls,^119^, ^120^, ^121^, ^122^ but other posttranslationally modified species of α‐synuclein have also been assessed.^123^ Further studies on larger and longitudinally characterized cohorts will be of great help to verify the reliability and reproducibility of these preliminary findings.

As an extremely intriguing possibility, seed amplification assays (SAAs) are novel ultrasensitive protein amplification methods able to detect protein aggregates in biological samples.^124^, ^125^ SAAs are already used with success for prion disease diagnostics and have now been implemented for identification of patients affected by synucleinopathy. In several works, SAAs showed excellent diagnostic performance in discriminating synucleinopathies from controls and from other neurodegenerative diseases since the prodromal disease stages.^126^, ^127^, ^128^, ^129^ Recently, pathological aggregates from patients with PD and MSA could be distinguished in CSF by using a combination of biochemical and biophysical methods on the products of α‐synuclein SAAs.^130^ SAAs have also been applied successfully in other biological matrices, such as olfactory mucosa and skin biopsies, revealing high diagnostic accuracy in discriminating MSA from tauopathies and PD from controls, respectively.^131^, ^132^ Further improvement in standardization and validation in different biological matrices is, however, required for the routine use of SAAs in clinical practice.^124^, ^133^

## 5 β‐Synuclein as a Candidate Biomarker for Synaptic Degeneration

β‐Synuclein has been assessed in a few studies as a body fluid biomarker for neurological disorders (Table 1). In 2016, the first quantitative data on CSF β‐synuclein concentrations were provided by using an innovative mass spectrometry approach. No difference was found in patients with PD compared with controls, but PD with dementia (PDD) and DLB cases showed slightly higher β‐synuclein values that became significantly increased when considering the β‐synuclein/α‐synuclein ratio. This suggested that β‐synuclein could be a potential biomarker of cognitive decline rather than of motor impairment in synucleinopathies.^108^ Among other neurodegenerative disorders studied, CSF β‐synuclein levels were found to be increased only in patients with CJD and AD.^108^, ^134^ These results on CSF β‐synuclein have been recently replicated with immunoassays, showing an excellent correlation with the antibody‐free, mass spectrometry method.^135^ Similarly to previous reports, only patients affected by AD and CJD, but not by synucleinopathies or other neurodegenerative disorders, showed increased CSF β‐synuclein levels.^135^

Overall, these findings indicate that CSF β‐synuclein alone is not a reliable diagnostic biomarker for synucleinopathies but, rather, might be a candidate biomarker for synaptic degeneration given its localization in presynaptic terminals.^135^ Moreover, the fact that β‐synuclein is not expressed in blood cells makes it even more promising as a peripheral blood biomarker in comparison with α‐synuclein and other synaptic proteins. For instance, neurogranin, which is one of the best characterized synaptic biomarkers, was repeatedly found to be elevated in the CSF of patients with AD, but similar changes were never observed in blood, probably because of the additional synthesis of this protein outside the CNS.^136^ On the other hand, β‐synuclein is only expressed in central neurons, and its blood levels more likely reflect synaptic degeneration. Accordingly, higher β‐synuclein levels were found in the serum of patients with AD compared with healthy controls and other neurodegenerative diseases.^134^ This finding has been confirmed in two validation cohorts.^134^

γ‐Synuclein has been poorly investigated as a fluid biomarker for neurological disorders. CSF levels of γ‐synuclein were measured in only a single study in patients affected by neurodegenerative diseases by quantitative mass spectrometry and showed increased levels in AD and CJD.^108^ Interestingly, γ‐synuclein is currently being tested as a potential fluid biomarker in brain and peripheral tumors.^137^

## 6 Use of Synucleins as Biomarkers in Pharmacological Trials

In clinical trials for disease‐modifying drugs, reliable biomarkers reflecting the underlying pathological process may help to enroll more homogeneous cohorts of patients and provide objective measures to better assess target engagement and outcome profile, thus improving the quality and the applicability of the results.^138^ CSF α‐synuclein has been used in clinical trials as an outcome measure, but the results are not yet available for all of them (Table 1).

Nilotinib is a protein kinase inhibitor being tested for PD and DLB to enhance intracellular degradation of α‐synuclein via ALP.^139^ In a clinical trial of efficacy, safety, and tolerability of nilotinib, 12 patients with PDD/DLB were randomized to receive either 150‐mg or 300‐mg nilotinib for 6 months. CSF was obtained at baseline and after 2 and 6 months of treatment. With respect to baseline levels, CSF t‐α‐synuclein was reduced at 2 and 6 months only in the 150‐mg dosage group.^140^ In a following trial of the pharmacokinetics and pharmacodynamics of nilotinib, 75 patients with PD were randomly assigned into five groups that received a single daily dose of placebo or 150‐, 200‐, 300‐, or 400‐mg nilotinib, with CSF collected 1 to 4 hours after drug administration. Nilotinib has been shown to enter the CNS in a dose‐independent manner, and a single 200‐mg dosage appeared optimal for impacting CSF biomarkers, including DA metabolites and α‐synuclein. However, no changes were found in CSF t‐α‐synuclein, whereas o‐α‐synuclein levels were significantly reduced in the 400‐mg group 3 hours after administration.^141^ In a more recent trial of KM‐819, a Fas‐associated factor 1 inhibitor, in healthy volunteers, CSF samples were collected at baseline and 7 days after the last drug administration, and no changes were observed in CSF o‐α‐synuclein.^142^ A randomized, placebo‐controlled study evaluating the safety and tolerability of bosutinib, another protein kinase inhibitor, in DLB started in 2019, and both plasma t‐α‐synuclein and o‐α‐synuclein were used as outcome measures (ClinicalTrials.gov identifier NCT03888222). Similarly, CSF and plasma levels of t‐α‐synuclein and o‐α‐synuclein are being used in an ongoing clinical trial of K0706, a tyrosine kinase inhibitor, in patients with DLB (ClinicalTrials.gov identifier NCT03996460) (Table 1).

The dual behavior of CSF t‐α‐synuclein as a marker of synucleinopathy and synaptic derangement poses relevant challenges for interpreting changes of its concentrations as an outcome measure and for verifying target engagement. To overcome these issues, CSF α‐synuclein species could be combined with other synaptic biomarkers, such as β‐synuclein, to better picture the synaptic dysfunction without being biased by α‐synuclein pathology. Furthermore, given the promising results obtained in AD and prion disease, β‐synuclein could be also used alone as a reliable outcome measure for clinical trials assessing novel drug candidates for preserving synaptic integrity.^135^

So far, CSF t‐α‐synuclein has never been considered among the inclusion criteria for enrolling patients because of its low diagnostic performance in discriminating synucleinopathies from other neurodegenerative disorders. In this scenario, α‐synuclein SAAs could be a reliable diagnostic test for the selection of patients thanks to their efficacy in identifying and partially differentiating synucleinopathies.^130^ SAAs are primarily designed to produce a dichotomous result about the presence or absence of pathological α‐synuclein, but because the seeding profiles depend on the amount of aggregated α‐synuclein in biological samples, they might be suitable for monitoring the effects of drugs countering α‐synuclein pathology.

The application of α‐synuclein and β‐synuclein as fluid biomarkers for clinical research is thus more than welcome, especially in association with more traditional markers including clinical scores and brain imaging. Their use in research would add valuable information with implications for patient selection and outcome monitoring in clinical trials. Nonetheless, several methodological issues, such as the standardization of quantitative procedures, should be addressed before these biomarkers can be used routinely in clinical practice.

## 7 Concluding Remarks and Future Perspectives

Most of the research on synucleins has so far mainly focused on α‐synuclein pathophysiology in synucleinopathies. In recent years, we have begun to broaden our view to the whole synuclein family to define their physiological role in neurons, characterize their pathological involvement in neurological disorders, and most important, develop new diagnostic and therapeutic tools for such conditions.

At a physiological level, α‐synuclein and β‐synuclein seem to act as housekeeping proteins in presynaptic terminals that regulate synaptic homeostasis and transmission. However, they might have wider activities in neuronal and nonneuronal populations. At a pathological level, although α‐synuclein is well known to aggregate, the modulatory properties attributed to β‐synuclein are still not clear. On one hand, β‐synuclein can mitigate α‐synuclein‐induced toxicity when coexpressed in certain conditions. On the other hand, β‐synuclein also has an aggregation potential that can cause neurodegenerative features similar to those described for α‐synuclein. It is possible that β‐synuclein can interfere with the aggregation dynamics of α‐synuclein but, at the same time, maintains the potential to aggregate under the appropriate conditions. In addition, β‐synuclein‐positive inclusions have been found in brain areas associated with cognition in patients with synucleinopathies and other neurodegenerative disorders, even without colocalizing with α‐synuclein aggregates. Whether these features have an impact in vivo is still an open question. Although very recent and not numerous, investigations on CSF indicate that β‐synuclein concentrations do not reflect an underlying synucleinopathy but, rather, may relate to ongoing synaptic degeneration. In this view, the association with β‐synuclein could improve the diagnostic performance of α‐synuclein given the dual behavior of the latter as a biomarker of synucleinopathy or of synaptic derangement. In addition, because β‐synuclein is specifically expressed in neurons, both its CSF and blood concentrations might be reliably applied as a surrogate biomarker of synaptic damage in neurodegenerative disorders other than synucleinopathies, such as AD. The use of α‐synuclein and β‐synuclein as biomarkers in research settings and routine clinical practice requires further investigations to overcome methodological issues concerning their measurements, but the results so far are encouraging. Our knowledge about synuclein pathophysiology adds greater insight into the neurobiology of synucleinopathies and allows us to better understand the strengths and limitations of their potential application as fluid biomarkers.

## Author Roles

(1) Research Project: A. Conception, B. Organization, C. Execution, D. Created the Figure; (2) Manuscript: A. Writing of the First Draft, B. Review and Critique.

L.B.: 1B, 1C, 1D, 2A

F.P.P.: 1B, 2A

G.B.: 1B, 2A

L.G.: 1D, 2B

S.H.: 2B

P.O.: 2B

M.O.: 2B

L.P.: 1A, 1C, 2B

## Financial Disclosures

G.B. is currently supported by the JPND bPRIDE (blood Proteins for early Discrimination of dEmentias) project. The Project leading this result has received funding under the call “JPco‐fuND‐2: Multinational research projects on Personalised Medicine for Neurodegenerative Diseases” (CUP number J99C18000210005). L.P. received research support from Fujirebio. P.O. received research support from The Michael J. Fox Foundation for Parkinson's Research (Grant MJFF‐010349) and Alzheimer Forschung Initiative e. V. (20059CB). M.O. was supported by grants from the German ministry of science and technology (FTLDc, moodmarker, Genfi‐Prox), the ALS association, the Thierry Latran foundation and Boehringer Ingelheim University Ulm institute. M.O. gave scientific advice to Biogen, Roche, and Axon Neuroscience. The other authors have nothing to disclose.

## Data Availability

Data sharing is not applicable to this article as no new data were created or analyzed in this study.
